# Assessment of medical students’ learning and study strategies in self-regulated learning

**Published:** 2016-04

**Authors:** ZAHRA JOUHARI, FARIBA HAGHANI, TAHEREH CHANGIZ

**Affiliations:** 1 Student Department of Medical Education Research Center, Isfahan University of Medical Sciences, Isfahan, Iran;; 2 Department of Medical Education Research Center, Isfahan University of Medical Sciences, Isfahan, Iran;

**Keywords:** Learning, Medical student, Assessment

## Abstract

**Introduction:**

Research on medical students shows that adopting self-regulation of effort, time, and study strategies can positively influence academic achievement. The purpose of the current study was to assess learning and study strategies in medical students.

**Methods:**

This cross-sectional study was carried out in 2014-2015 at Isfahan University of Medical Sciences. The sample size was determined 360 students based on the results of a pilot study on 30 members of the study population. Medical students in the first to fourth year of their studies were selected through simple sampling randomly. A valid and reliable Persian translation of Learning and Study Strategies Inventory (LASSI) questionnaire was completed by the students. It measures three latent factors of self-regulated learning: Skill, Will, and Self-Regulation. It consists of 80 questions in ten different scales (each scale including eight questions and a variable score of 8-40). Data were analyzed using t-test, correlation analysis, and ANOVA.

**Results:**

Considering the ten LASSI scales, the highest mean score belonged to test strategies (28.67±4.44), and the lowest mean to self-testing (21.91±4.91). The results showed significant statistical differences between male and female students in selecting the main idea, attitude, and self-testing. ANOVA and post hoc Tukey tests showed a significant difference between the mean scores of different areas of LASSI among students with different grade point average (GPA) in the university. In all areas except the study aids, the mean scores of students with GPA higher than 17.5 were significantly higher than those of students with GPA lower than 14.5.

**Conclusion:**

The results showed that students need help and consultation in most areas of learning and study strategies. Using 10 areas of LASSI can determine the strengths and weaknesses of students in various areas. Knowing their own limitations, students will be able to improve their study habits. Hence, it is suggested to evaluate the students when enrolling at universities and design educational programs based on the students’ characteristics.

## Introduction

Self-regulated learning (SRL) is an important concept in higher education research (1). SRL is considered as a necessary requirement of life-long learning (2, 3). Self-regulated learners are more effective in learning and have a repertoire of learning and study strategies to match different situations (4). Furthermore, the self-regulated use of learning strategies helps students to take responsibility for their own learning (5).

Medical students must use key self-regulating skills in clinical courses that involve specialized aspects such as dealing with patients, meaningful learning, use of critical thinking, self-assessment of activities, and need for updating personal information (6).Several meta-analysis studies have demonstrated the significance of SRL and its underlying components for students' academic achievement (7, 8). Research on medical students shows that adopting self-regulation of effort, time, and study strategies can positively influence academic achievements (9). West and Sadoski confirmed that study strategies, especially self-testing, are strong predictors of medical students’ grades in the first semester of medical school (10). Dunn studied pathophysiology students and found that, despite the difficulty of the course, the use of self-regulated learning strategies had positive results for both students and instructors (11). Medical schools aim to graduate as medical doctors who are able to self-regulate their learning (12).

Learning strategies include thinking, behavior, opinions, or emotions that facilitate receipt, understanding, and subsequent transfer of new knowledge and skills (13). Learning and study strategies are important factors in academic achievement (14, 15). Several studies have demonstrated the link between positive academic behaviors and grade point average (GPA) obtained in university (16-18). One of the most common scales used in some studies that measure self-regulation in learning is the learning and study strategies inventory (LASSI) (19, 20). It is currently used by almost 2000 tertiary institutions over the world. It was developed as a diagnostic tool to measure how students use learning strategies in academic environments, so they can be promoted through interventions. It is designed to gather information about learning and study practices and attitudes (13). The learning and study strategies inventory is widely used in assessing the students’ learning and study strategies at both high school levels and university (21). Self-assessment is recognized as a necessary skill for lifelong learning (22). Students use LASSI for self-assessment in their study strategies.

The data from LASSI can be used by teachers and students to improve teaching and learning (23). A study in Hong Kong showed that the integration of LASSI with the information system of a university was useful as students had the possibility of reviewing their progress in terms of learning. Also, teachers can design appropriate teaching and learning activities according to the strengths and weaknesses in the students' learning (24). The results of Ahmadi's study showed that many domains of LASSI among medical and dental students were low (25). Hence, the first step for any intervention is the assessment of the students’ study and learning skills. The current study aimed to assess learning and study strategies in medical students so that educators and students are assisted in self-regulated learning and the related skills.

## Methods

The present cross-sectional study was carried out in 2014-2015. The study population consisted of all medical students in their first to fourth years of study at Isfahan University of Medical Sciences. The sample size was determined 360 based on the results of a pilot study on 30 members of the study population. At Isfahan University, students of each year are divided into two even and odd classes randomly; one class was selected using simple random sampling from each year to answer the questionnaires. In the selected classes, all students received the questionnaire (a total of 400 questionnaires). 

Data were gathered using the second edition of Learning and Study Strategies Inventory (LASSI) (26). Designer of LASSI originally proposed that it measures three latent factors of self-regulated learning: 1. Skill (subgroups of information processing, selecting main idea, and test strategies), 2. Will (subgroups of anxiety, attitude, and motivation), and Self-Regulation (subgroups of concentration, self-testing, study aides, and time management (18, 27). Psychometric properties of LASSI have been examined in several studies, showing good validity and reliability (13, 19). It consists of 80 questions in 10 different scales (each scale including eight questions and a variable score of 8-40). The LASSI yields ten individual scale scores, one for each of the ten scales. No total score is computed because this is a diagnostic instrument (26). It provides assessment in ten learning and studying scales as follows:

**Selecting Main Ideas scale**: Students’ scores on this scale measure their skills at selecting important information.

**Information Processing scale**: Students’ scores on this scale measure how well they can create imaginary and verbal elaborations and organizations to foster understanding and recall.

**Test Strategies scale**: Students’ scores on this scale measure their use of test taking and test preparation strategies.

**Anxiety scale**: Students’ scores on this scale measure how tense or anxious they are when approaching academic tasks

**Attitude scale**: Students’ scores on this scale measure their general attitudes and motivation for succeeding in school and performing the tasks related to school success.

**Motivation scale**: Students’ scores on this scale measure the degree to which they accept responsibility for performing the specific tasks related to school success.

**Time Management**: Students’ scores on this scale measure the degree to which they create and use schedules.

**Self-testing scale**: Students’ scores on this scale measure their awareness of the importance of self-testing and reviewing and the degree to which they use these methods.

**Study Aids scale**: Students’ scores on this scale measure their ability to use or create study aids that support and increase meaningful learning and retention.

**Concentration scale**: Students’ scores on this scale measure their abilities to concentrate and direct their attention to school and school-related tasks, including study activities.

The questions were scored based on a five degree scale from 1 to 5 (from "not at all like me" to "very much like me"). Weinstein in the 2002 edition of this inventory calculated the reliability of the questionnaire using Cronbach's alpha 0.71-0.86 for ten areas (13). For the present study, Cronbach's alpha was calculated 0.71-0.89 for ten areas. Also test–retest reliability with an interval of 3 to 4 weeks was conducted and a coefficient of 0.85 was obtained the whole scale method. The face validity of the questionnaire was investigated and confirmed by five experts in the field of medical education and education planning.

In order to distribute the questionnaires, some university employees were sought for help. They were given enough information to provide the students with necessary information and obtain their consent. The students’ participation in the study was voluntary, and after receiving the questionnaire, they could leave the study at any time. Questionnaires were filled out using self-administered method. The time given for filling each questionnaire was 20-25 minutes. The questionnaires were collected at certain locations in the classes and at the university hospital. Each participant was given a small gift, to be appreciated, after returning the questionnaire. 

Mean scores for different areas of LASSI were compared with the students’ GPA. For this purpose, the students were divided into three groups based on mean and standard deviation of GPA obtained in university, score for the low GPA (below 14.5), the middle GPA (between 14.5 to17.5), and the high GPA (17.5 to 20). Also, the median scores of ten areas were compared with the median normative score of the students in the United States. The results clearly show the students their strengths and weakness in areas related to strategies learning. Students who have scores below the 50th percentile need guidance and support in learning and study strategies. Students who have scores at 50 to 75 percentile of the normative scores are showing good skills in study and scores above 75 percentile indicate higher skills the students use as to the learning and study strategies (13). The data were analyzed using SPSS 18 software. The significant P value was considered 0.05. Descriptive analysis of the data was carried out using mean, standard deviation, and frequency. In order to compare the quantitative data and determine the correlation of factors, independent t-test, correlation, analysis of variance (ANOVA) and post Hoc Tukey tests were used.

## Results

Three hundred and sixty three students returned the questionnaires filled (return rate of 91%). The demographic characteristics of the medical students participating in the study are shown in Table 1. The mean and standard deviation of the students' GPA was 16.03±1.40 (minimum 12 and maximum 19.60); 72.7% (264) in basic medicine and 27.3% (99) in physiopathology period. Only 20.9% (73 students) had formal training in study methods and self-regulation. The highest mean value belonged to the test strategies (28.67±4.44) while the lowest mean belonged to self-testing (21.91±4.91) (Table 1). Learning and study strategies inventory (LASSI) scores in each 10 scale**s** are summarized in Table 2.

**Table 1:** Demographic characteristics of medical students participating in the study

**Items**		N (%)
**Sex**	Female	200 (55.1%)
	Male	163 (44.9%)
**Marriage**	Single	349 (0.96%)
	Married	14 (0.4%)
**Education**	Yes	73 (20.9%)
	No	290 (79.1%)
**Lodging**	Dormitory	160 (44.8%)
	With family	203 (55.2%)
**GPA**	<14.5	31 (14.0%)
	14.5-17.5	153 (68.9%)
	>17.5	38 (17.1%)

**Table 2:** Learning and study strategies inventory (LASSI) scores in all 10 scales

	Items	Min	Max	Med	Mean±SD
**Skill**	Test strategies	14	40	29	28.67±4.44
	Information processing	8	40	26	26.20±4.64
	Selecting the main idea	12	40	29	28.47±4.54
**Will**	Motivation	11	40	26	26.22±4.87
	Attitude	13	38	28	27.94±4.77
	Anxiety	9	40	28	27.56±6.01
**Self-regulated**	Concentration	10	40	26	25.96±5.54
	Study aids	8	35	22	21.97±4.32
	Self-testing	9	40	22	21.91±4.91
	Time management	8	40	24	24.37±5.85

In order to investigate the differences between learning and study strategies among male and female students, independent t-test was employed. The results showed significant statistical differences between male and female students in the areas of selecting main idea, attitude and self-testing. Learning and study strategies inventory (LASSI) scores in each 10 scales based on sex and habitat are shown in Table 3. Also investigating the mean scores of different areas using t-test only showed a significant difference between the mean score of concentration between students with formal training (27.58±5.45) and those without formal training (25.56±5.50) (p=0.006). In other words, students with formal training on learning strategies had a higher mean of concentration.

**Table 3:** Learning and study strategies inventory (LASSI) scores in all 10 scales based on sex and habitat

**Items**	**Male**	**Female**	**p**	**Dormitory**	**Private home**	**p**
	**Mean±SD**	**Mean±SD**		**Mean±SD**	**Mean±SD**	
**Test strategies**	29.07±4.50	28.27±4.40	0.09	29.28±4.35	28.00±4.46	0.007*
**Information processing**	26.33±4.98	26.03±4.22	0.54	26.46±4.89	25.80±4.34	0.181
**Selecting the main idea**	28.95±4.51	27.89±4.55	0.02*	29.01±4.34	27.90±4.73	0.02*
**Motivation**	26.18±5.11	26.29±4.60	0.84	26.46±5.05	25.90±4.67	0.29
**Attitude**	28.59±4.96	27.22±4.45	0.008*	28.42±5.08	27.40±4.34	0.04*
**Anxiety**	27.18±6.21	28.03±5.78	0.18	27.86±5.97	27.38±6.10	0.45
**Concentration**	26.13±5.49	25.75±5.69	0.52	26.52±5.81	25.33±5.17	0.04*
**Study aids**	21.77±4.37	22.33±4.26	0.22	22.27±4.69	21.57±3.85	0.137
**Self-testing**	21.31±4.84	22.63±4.95	0.01*	21.91±5.17	21.81±4.55	0.84
**Time management**	23.91±5.82	24.89±5.93	0.12	24.16±5.81	24.66±6.04	0.43

*Significant

Comparing the mean scores of different areas between students living in dormitories and other students showed that the students’ scores living in dormitories regarding test strategies, selecting main idea, attitude, and concentration are significantly lower than that of other students (Table 3). Also, comparing the mean scores of married and single students showed no significant difference. In addition, there was a significant and positive relationship between all areas of LASSI and students' GPA (from 0.16 to 0.38 p> 0.05), except for the area of study aids.

ANOVA and follow-up Tukey test showed a significant difference between the mean scores of students with different GPA with respect to different areas of LASSI. In all areas, except for the study guide and selecting the main idea, the mean scores of students with GPA higher than 17.5 were significantly higher than those of students with GPA lower than 14.5. Learning and study strategies inventory (LASSI) scores in each 10 scales among different levels of GPA students are summarized in Table 4.

**Table 4:** Learning and study strategies inventory (LASSI) scores in all 10 scales in different levels of GPA students

** **	**Items**	**GPA Averages**			
		**Level 1** **<14.5**	**Level 2** **14.5-17.5**	**Level 3** **>17.5**	**p**
**Skill**	Test strategies (TST)	26.60±4.41	28.91±4.07	30.05±3.83	0.002***
	Information processing (INP)	23.90±4.84	26.48±4.37	27.11±3.65	0.005***
	Selecting the main idea (SMI)	27.66±4.44	28.64±4.71	29.27±3.74	0.354
**Will**	Motivation (MOT)	23.59±4.19	26.18±4.79	29.27±4.39	0.001*****
	Attitude (ATT)	26.83±4.68	27.83±4.42	30.38±4.10	0.002***
	Anxiety (ANX)	27.17±7.30	28.13±5.98	30.76±4.48	0.027**
**Self-regulated**	Concentration (CON)	24.66±5.30	25.93±5.23	28.57±6.42	0.009**
	Study aids (STA)	20.62±4.43	21.73±4.24	22.38±3.48	0.230
	Self-testing (SFT)	20.00±5.41	21.41±4.52	22.92±5.65	0.049**
	Time management (TMT)	21.00±5.31	24.35±6.21	27.32±5.30	0.001*****

* Significant in level 1 with level 2

** Significant level 1 with level 3

*** Significant level 1 with levels 2 and 3

**** Significant in levels 1 and 2 with level 3

***** Significant in three levels different

Also, The students’ median scores regarding ten areas of LASSI were compared with the normative median score of the students in the United States and the results are shown in Table 5.

**Table 5:** Comparison of the students’ median scores of LASSI with the normative median score of students in the United States

**Percentile**	**TST**	**SFT**	**STA**	**SMI**	**INP**	**CON**	**ANX**	**TMT**	**MOT**	**ATT**
**99**	40	40	38	40	40	40	40	40	40	40
**95**	38	36	35	38	38	37	37	37	39	39
**90**	36	33	33	37	35	35	35	35	38	-
**85**	35	31	32	35	34	34	33	33	37	38
**80**	34	30	30	34	33	33	32	32	36	37
**75**	33	29	29	33	31	32	31	31	-	-
**70**	32	28	-	32	30	31	30		35	36
**65**	-	27	28	31	-	30	29	30	34	-
**60**	31	26	27	30	29	29	28*	29	33	35
**55**	30	-	26	29*	28	-	27	27	-	-
**50**	-	25	-	-	27	28	26	-	32	34
**45**	29*	24	25	28	-	27	25	26	31	-
**40**	28	23	24	27	26*	26	24	25	-	33
**35**	-	22*	-	26	25	25	23	24*	30	-
**30**	27	-	23	25	24	24	22	23	29	32
**25**	26	21	22*	24	-	23	21	22	28	-
**20**	25	20	21	23	23	22	20	21	27	31
**15**	24	19	20	22	22	21	18	20	26*	30
**10**	23	17	19	21	21	19	17	18	24	28*
**05**	21	15	17	18	19	17	14	16	22	26
**01**	18	12	13	13	15	13	10	12	18	21

* Students’ median scores of LASSI in Isfahan University

## Discussion

This study was carried out in order to investigate study strategies among medical students of Isfahan University of Medical Sciences in the year 2014-2015. Study strategies in students’ learning were investigated using Weinstein’s 10 areas of LASSI. The highest mean score belonged to the area of test strategies with the area of selecting the main idea being a close second. The results showed that due to direct and tangible effect of these two areas on academic achievements, students pay more attention to these two areas. Also, most guiding organizations emphasize these two areas for academic achievement (27, 28). The results showed that the scales of self-testing and study aids had the lowest and second lowest average scores, respectively. Self-testing shows the ability of students in assessment of their work while study aids helps the students use various available guides to improve their learning. These results can be related to the students’ attitude on the effectiveness of these areas on their academic achievement (29).

Kombi found that only 45.5% dental students used self-testing after reading a textbook chapter (30). A study by Stewart on pharmacology students showed that students with better self-testing abilities are more successful in their final exams (31). Other studies show that incorrect study habits of students need to be identified and fixed because many of these incorrect habits get transferred to higher education levels. Therefore, it is necessary to provide suitable guidance to improve these incorrect habits (32, 33).

The results showed a statistically significant difference between the mean scores of male and female students. The female students had higher mean scores in the areas of selecting the main idea and attitude and the male students had higher mean scores in self-testing scale. A study in Iran showed that female students use the test strategies and selecting the main idea more than the male students (25). Results of a study in Brunei showed that female students were more effective users in test preparation and concentration on study strategies (34). Also, students in later years made more use of learning strategies (35).

The findings showed that there was a significant difference between the mean scores of students in different years regarding the area of attitude, with the mean scores being lower in later years. This result is similar to the study reported by Ahmadi on the students of Shahed University (25), showing that, assuming also the initial assessment of students' skills, a constant observation is necessary to maintain a suitable level in this area. The results of a study in Hong Kong also showed a significant difference between learning strategies of students in their first year and later years, demonstrating that the attitude and motivation of students in their later years play an important role in their use of learning strategies (35).

A study conducted in Shiraz showed a positive relationship between motivation and self-regulation among students. It also showed a negative relationship between self-regulation and anxiety (36). Another study in Turkey showed a direct association between positive attitude and use of learning strategies (37). Studies show that, in addition to considering the factors that directly affect learning, other factors such as planning and general organizing, outside regulations, environmental restoration, recall, comprehension, and cognitive and metacognitive factors can also affect the use of learning strategies (38). Flowers, in his study on the content validity of LASSI, considered LASSI a suitable tool for teachers and consultants at universities (39).

Investigating the education history of the participants related to their study and learning strategies showed that students with previous education regarding study and learning strategies had higher concentration. The results of a study on medical students showed that attending study skill workshops and learning related abilities can empower the students in the areas of selecting the main idea, study aids, information processing, self-testing, and use of test strategies (40, 41). However, teaching self-regulation strategies and practicing them in class can create opportunities that help the students manage and monitor their learning (42).

Comparing the mean scores of different areas among the students with different GPA showed that those with higher GPA scores had better self-regulation in using learning strategies. Several studies have demonstrated a meaningful difference between the students with high and low academic achievements in terms of using learning strategies (4, 27).

Comparison of the study scores of ten areas with the normative scores of students in the United States showed that except for the area of selecting the main idea and anxiety control, the participants scored lower than 50 percentile of the students in the United States. Based on the statistical analysis, these students require consultation. Comparing the scores of prospective students at University of Isfahan and the study participants indicated that, except for the area of selecting the main idea, prospective students of University of Isfahan had higher scores compared to the participants in this study (41).

Several studies have shown a relationship between the students’ scores with respect to learning strategy questionnaires and their performance in academic tasks. The results of the study by West showed that time management and self-testing are strong predictors for the success of medical students in their first year (10). Also, a study by Loub on pharmacology students showed that anxiety, concentration, selecting the main idea, and exam strategies had positive correlations with the success of freshman students (17). Haught showed that students who personally received the results of their LASSI questionnaire after a certain period of time revealed a significant increase in their scores in the areas of selecting the main idea, attitude, motivation, anxiety, time management, concentration, and exam strategies (43). Noting the limitations of the current study, one can point to the study’s focus on a single university and solely investigating basic medicine and physiopathology students.

## Conclusion

The findings showed that students need help and consultation in most areas of learning strategy. Medical education is a lifelong process, and medical students must be lifelong learners. Medical education burdens a great bulk of information with limited time. Therefore, the focus of medical education should be on the learner facilitating the learning process. Using 10 LASSI areas can determine the strengths and weaknesses of students in various areas. Awareness about their own weaknesses, students will be able to improve their study habits. Education planners and educators can design more suitable education programs through knowing the characteristics of their students. Thus, every student is recommended to be evaluated when enrolled at university, and the education program needs to be designed based on the characteristics of students. Also, given the length of medicine course and changes in the areas of study strategies throughout the study years, it is necessary to constantly monitor and evaluate the characteristics of students in order to provide the necessary strategies for improving the students’ habits as required. 

## Footnotes

Authors and contributors: The idea for the study was developed by ZJ, FG, and T CH. Zahra Jouhari drafted the manuscript. Fariba Hahgani as the supervisor participated in the study design, supervised the data collection and data analysis process, and revised the manuscripts. Taherh Changiz as the research consultant participated in the study and advised during the study.
